# Cost-effectiveness of a novel smartphone application to mobilize first responders after witnessed OHCA in Belgium

**DOI:** 10.1186/s12962-020-00248-2

**Published:** 2020-11-17

**Authors:** Steven Vercammen, Esther Moens

**Affiliations:** 1 EVapp vzw, AA Tower – 8th floor, Technologiepark 122 (zone C2a), 9052 Zwijnaarde, België; 2 UGent, Sint-Pietersnieuwstraat 25, 9000 Gent, Belgium

**Keywords:** OHCA, Public AED, Cost-effectiveness, Mobile phone application, I18, Government Policy, Regulation, Public Health

## Abstract

**Background:**

EVapp (Emergency Volunteer Application) is a Belgian smartphone application that mobilizes volunteers to perform cardiopulmonary resuscitation (CPR) and defibrillation with publicly available automatic external defibrillators (AED) after an emergency call for suspected out of hospital cardiac arrest (OHCA). The aim is to bridge the time before the arrival of the emergency services.

**Methods:**

An accessible model was developed, using literature data, to simulate survival and cost-effectiveness of nation-wide EVapp implementation. Initial validation was performed using field data from a first pilot study of EVapp implementation in a city in Flanders, covering 2.5 years of implementation.

**Results:**

Simulation of nation-wide EVapp implementation resulted in an additional yearly 910 QALY gained over the current baseline case scenario (worst case 632; best case 3204). The cost per QALY associated with EVapp implementation was comparable to the baseline scenario, i.e., 17 vs 18 k€ QALY^−1^.

**Conclusions:**

EVapp implementation was associated with a positive balance on amount of QALY gained and cost of QALY. This was a consequence of both the lower healthcare costs for patients with good neurological outcome and the more efficient use of yet available resources, which did not outweigh the costs of operation.

## Background

Out-of-hospital cardiac arrest (OHCA) is a major health problem, with Belgian incidence rates of emergency medical services (EMS) attending OHCA of cardiac origin of approximately 82.8 per 100 000 person-years (86.4 on average in Europe) [1–3]. Important determinants of survival after OHCA are early cardiopulmonary resuscitation (CPR) as part of basic life support (BLS) and rapid defibrillation to restore spontaneous circulation [4, 5]. Delayed arrival time of the EMS has been associated with poor survival [6, 7]. Publicly available automated external defibrillators (AED) permit bystanders of OHCA or first responders, not trained in advanced life support (ALS), to provide early defibrillation prior to EMS arrival. A recent meta-analysis concluded that bystander AED use was associated with increased survival to hospital discharge (all rhythms OR: 1.73, shockable rhythms OR: 1.66) and favourable neurological outcome (all rhythms OR: 2.12, shockable rhythms OR: 2.37) [8]. In recent studies, high survival rates of 30–70% have been reported for OHCA patients defibrillated with a publicly accessible AED, early after collapse [9–14]. No associations were found between bystander AED use and favourable neurological outcome in case of cardiac arrest with non-shockable rhythm (OR: 0.76) [8]. Although some studies could not find any survival benefit from bystander defibrillation, comprehensive analysis of the data showed limitations such as very high median response time before arrival (> 10 min) [15] or a limited part of patients effectively resuscitated by public AED [16]. Larsen et al. modelled the ‘Chain of Survival’ concept [17], describing the effect of the time delay of access to CPR, defibrillation and ALS on survival as: survival = 67–2.3% minutes of delay of CPR-1.1% minutes of delay of defibrillation-2.1% minutes of delay of ALS [18]. The model is still particularly interesting to compare survival for different scenarios, similar outside the response time of the interventions. Previous studies have been conducted on the clinical benefits and cost-effectiveness of public access defibrillation (PAD) programs, aimed at increasing the use of AED, prior to EMS-arrival [19, 20]. Comparison of such programs is challenging, considering the high degree of heterogeneity in the types of programs implemented (e.g., static vs mobile AED use, strategy for activation, defibrillation by professionals, the public, or combinations of both) and the context within which they operate (baseline availability of public AED, overall chain of survival and level of care, geographic variations, socio-economic disparities) [20, 21]. Comparison is even more hampered by the large differences in interpretation of the Utstein definitions [22, 23] for standardized reporting on OHCA [24]. Although ‘bystander CPR' is defined, in scenarios that included community-response systems, firefighters and/or police personnel, the percentage of agreement that 'bystander CPR' had been performed ranged from 16–91% [24]. Nevertheless, previous civilian-based resuscitation initiatives, activating volunteers via a mobile phone application [25–29], have underscored the importance of increased rates of early BLS and/or use of public AED, even achieving survival to discharge rates of 40–50% [28].

**Fig. 1:**
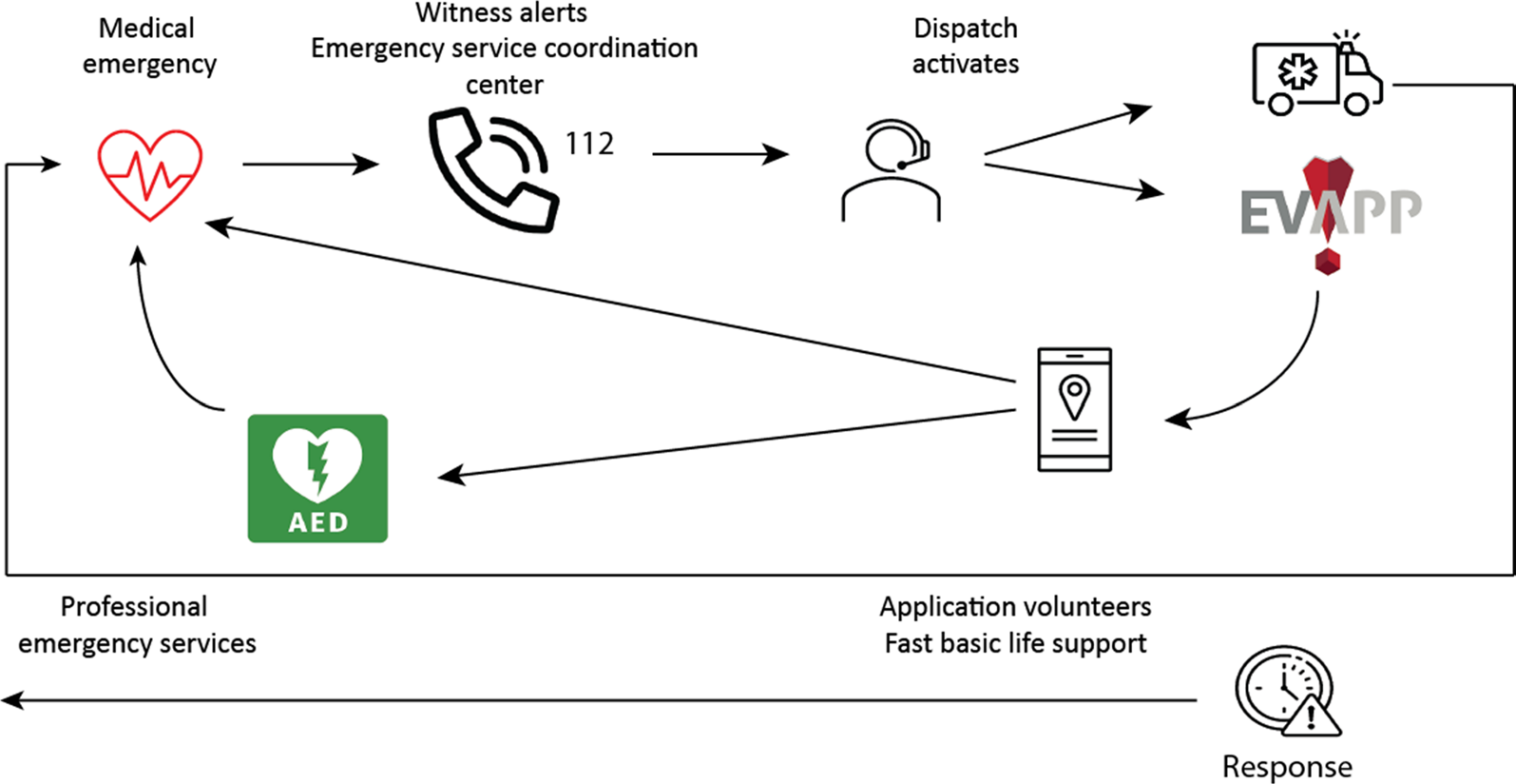
Schematic representation of the EVapp-system

In Belgium, EVapp is the first civilian-based initiative to increase survival after OHCA. The system is developed as an application for smartphones, based on the Android and iOS operating system, and connected with the operating software of EMS dispatchers in Belgium. EVapp maintains a database of qualified volunteers and an up-to-date overview of the publicly available AED. Publicly available AED in Belgium are registered in the Federal Public Service Database and professionals as well as lay responders are authorized to use an AED by law in patients with OHCA [2]. In case of an alert for OHCA at the 112-dispatch centre, EVapp volunteers are activated in parallel and at the same time of dispatchment of the EMS. The application compares the geographical position of all volunteers and the location of the registered public accessible AED to the position of the incoming emergency call of the suspected cardiac arrest. Activation is based on two principles: (1), the two closest volunteers are mobilized towards the victim, (2), the third and fourth closest volunteers are send to the victim via the closest public AED. The fifth closest volunteer is again send directly to the victim (Fig. 1). The application is programmed in such a way that volunteers present within a radius of 500 m from the suspected arrest first receive a warning alert. The smartphone application provides guidance to the geolocations of the suspected cardiac arrest and/or AED. If the desired amount of responses (> 3) within 500 m is not met, the radius is systematically increased to a maximum of 1500 m. In case no volunteers are found, not only their current geolocation is considered, but also their home and/or work addresses can be used for activation, as these addresses may imply a relatively high probability of being in the neighbourhood (static users). Those EVapp volunteers will also receive an alert on their mobile phone via a text message, which they can choose to accept or decline in case their 4G would not been activated.

There is no immediate validation process implemented to evaluate if the volunteers that accepted the alert effectively reach the victim. Yet, the fact that 5 volunteers are alerted provides a margin of safety to avoid such situations. During the course of EVapp activation, performance parameters are being automatically registered by the software (Table 1). After each intervention, the volunteers receive a feedback form. This form offers to share their experience about the technical performance of the application (SMS function and locations), about the course of the intervention, the resuscitation attempt and the interactions with other volunteers and professional care providers.

**Table 1:** Important performance parameters automatically registered during each EVapp activation and inquired from the mobilized volunteers through the application

Parameter	Unit
Automatic registration Time of EVapp activation at EMS dispatcher	Hour, minute, second
Time of volunteers detected within 1,5 km + time of alert accepted by volunteer	Hour, minute, second
Time of search for static users	Hour, minute, second
Course of the resuscitation EMS vs network initiated CPR and defibrillation?	Quantitative scoring + plain text
Competence of volunteers, AED availability, accurateness location and navigation, user-friendliness, psychological or medical info desired?	

People who can submit a certificate of qualification for BLS (minimal 3 h of training in the last 5 years) can volunteer in the network. Volunteers eligible for participation are initially recruited through interaction with professional organisations (firemen, health workers) and through the local departments of the Red Cross organisation. Individual permission to join the network is requested through the EVapp website (EVapp.org) or after downloading the app. An EVapp administrator verifies if each volunteer has provided a valid certificate of qualification for BLS. Upon positive evaluation, volunteers are added to the EVapp database. In addition, as soon as the city or municipality is interested in setting up a citizen assistance network, a local steering committee is assembled. The steering committee can help to support recruitment by providing promotional material or organising events. It was preliminary estimated that for Belgium, comparably to The Netherlands, a country with an extensive civilian-based network of BLS-skilled volunteers and public AED [28, 30, 31], approximately 1% of the total population should be included in the EVapp volunteer database. Such amount of volunteers corresponds to approximately 3.7 volunteers per km^2^ in Belgium [32].

The aim of the current study was to simulate the potential increase in annual survival from witnessed OHCA of cardiac origin, and the decreased costs per QALY, associated with a more efficient use of the yet existing resources (*i.e.* BLS-trained volunteers and public AED), upon nation-wide EVapp implementation in Belgium. Early validation was performed by use of the first data obtained from a pilot study of EVapp implementation in the rural area of Hoogstraten in Flanders. Positive projections in this study and further validation may convince local policy makers to implement EVapp in their city or municipality.

## Methods

### Setting and perspectives

An accessible model was developed to evaluate cost-effectiveness and survival gain after OHCA, associated with EVapp implementation in Belgium. The study is reported with consideration of the reporting standard for health-economic evaluations (CHEERS) [33].

Survival from OHCA of non-cardiac origin, *i.e.,* caused by an external agent, is very low, irrespective of the type and time of first response [34]. Early response moreover conservatively implies that the OHCA is witnessed. The target population in this study was therefore restricted to patients suffering from witnessed OHCA of cardiac origin in the Belgian population. With a total population of approximately 11.4 million people [32], this corresponded to around 6150 cases per year (approximately 86% of 11,000 cases were of cardiac origin, approximately 65% was witnessed) [2, 3].

The perspective of the study was the fact that early CPR and defibrillation, before the arrival of the EMS, would increase the amount of OHCA-survivors. This would lead to a total increase in the healthcare costs. It was prospected that the amount of survivors with good neurological outcome would increase ascendingly by the early intervention, meaning that overall a positive balance on the cost per QALY would be achieved.

Two different scenarios were directly compared, *i.e.,* an estimate of the current survival and costs related to the baseline interventions after OHCA alert in Belgium, and a presumed second scenario consisting of nation-wide EVapp implementation. Possible interventions for both scenarios were grouped into three categories *i.e.,* first response by either: (1), the emergency medical service (EMS), (2), bystanders or first-responders performing early CPR (present or activated), (3), bystanders or first-responders performing early CPR and defibrillation (present or activated). These scenarios are further referred to as ‘EMS’, ‘bCPR’ and ‘bCPR + AED’, respectively.

Healthcare related costs and productivity loss for OHCA-patients were evaluated in function of the neurological outcome, over a period of 6 years, i.e., the long-term mean survival after OHCA [35, 36]. Costs for EVapp implementation were included based on nation-wide implementation. Since EVapp aims to increase the use of available resources, major investments were generally not prospected.

### Outcomes and measurement of effectiveness

The main health endpoints evaluated in this research were survival and neurological outcome after OHCA, depending on the type (and time) of first intervention after the alert. As core outcomes, both 30 day survival or survival to hospital discharge were included, in accordance with the updated Utstein guidelines [23] for reporting on OHCA. The study on bystander use of static AED by the Belgian healthcare knowledge-centre was used as the primary source to document the baseline scenario [2]. This study provided analysis of registered data from the MUGREG/ SMUREG (2015), a second-tier unit staffed with emergency physicians and specialised nurses [37] and a re-analysis of the Belgian data of the European EuReCa ONE study [3]. Other data was obtained by focussed search in the Web of Science database, using the keywords ‘out of hospital cardiac arrest’ and ‘survival’. Limited studies were available that can fully represent the scenario of nation-wide EVapp implementation in Belgium. A selection of studies was made for which the setting of the study was conceivably comparable or relevant for Belgium (i.e., level of care, demography, geography, EMS response time), or in which values regarding outcome were obtained from meta-analysis. A large population-based OHCA registry demonstrated that early defibrillation contributed to improved 1 month survival [38], while other studies found differences in outcome depending on the shock provider [10, 14, 39, 40]. In this model, the main focus was on the time-delay of intervention, i.e., no distinction was made between first-responders or EVapp mobilized volunteers to perform CPR or defibrillation. The established model of Larsen et al. [18] was particularly useful as outcome estimator between the different scenarios, controlling all factors apart from time-delay. Using this model, the delay in ALS was approximated by the median reported arrival time of the MUGREG/SMUREG of 12 min [37] for all responses. Delay in CPR and defibrillation was approximated by the average ambulance arrival time of 10 min [41] for the ‘EMS’ response in Belgium. Delay in CPR was instead approximated by 5 min for the ‘bCPR’ and ‘bCPR + AED’ response. For the latter response additionally, the delay in defibrillation was approximated by 6.5 min for the ‘bCPR + AED’ response (objective EVapp: response within 5–8 min). The results of other studies were included in the survival estimations in case the comparator of the study (survival after EMS response) was comparable to the baseline case for Belgium (10% survival for EMS arriving within 10 min) or in case odds ratios based on extensive meta-analysis or literature review had been calculated [8, 10, 42]. Different studies reported very high average survival rates for bystander defibrillation (of shockable rhythms) of 35–70% [9, 11–14]. Such high survival rates may represent a realistic best case survival estimate in case the time of intervention (both CPR and defibrillation) indeed decreases dramatically (< 8 min) [43]. For the ‘EMS’ and ‘bCPR’ response, the median case was obtained as the average of the worst- and best case value in absence of a third relevant value. The fraction of survivors with good neurological outcome (*i.e.,* low cerebral performance category (CPC 1–2) [44]) after OHCA was around 85–92% in absence of community-based systems based on retrospective analysis of registries [20, 45, 46]. Based on meta-analysis, the odds ratio for favourable neurological outcome in case of bystander AED use was 2.1 for all rhythms and 2.4 for shockable rhythms [8]. When in this model the fraction of survivors with CPC 1–2 was evaluated at 85%, on average, the odds for good neurological outcome corresponded fairly with such ratios.

AED use by non-EMS personal in Belgium was reported in 7 of 105 OHCA cases (6.7%) [3], yet according to the (incomplete) data of the 2015 MUGREG/SMUREG registry report [37], this value was only around 0.22% (24/10,880) [2]. According to the EuReCa-one study, in cases where CPR was attempted, 47% on average was attempted by a bystander, with a range between 6.3% and 78% among countries [3]. The level of CPR-training in the Belgian general population is yet low to moderate [2]. The current fraction of bCPR in Belgium for witnessed OHCA was estimated at 33%. Based on recent initiatives to mobilize volunteers via mobile phone application [25, 26, 28, 47], responses of bCPR and bCPR + AED were assumed as 50 and 25%, respectively for the scenario of EVapp implementation.

As secondary outcome, QALYs were estimated over the average long-term survival after OHCA and by use of CPC-associated annual survival rates (0.9 for CPC 1–2 vs 0.8 for CPC 3–4) [20, 46]. The utility after OHCA had been rated as approximately 0.6–0.8 in general vs 0.8–0.9 for CPC 1–2 and 0.4 for CPC 3–4 [20, 48–52].

### Resources and costs

#### AED devices

It has been estimated that around 10,000 AED-devices are operational in Belgium*,* corresponding to 0.9 per 1000 inhabitants [2]. The devices are predominantly deployed by private actors (70%) [2]. At first instance, systematic investments in publicly available AED were not foreseen in the model to achieve the objectives of early defibrillation (< 8 min post collapse). This assumption needs to be further validated.

#### Patients productivity loss

The mean age at OHCA was approximately 65 years [2, 3, 37], which is around the pension age for most employees in Belgium [53]. Total productivity loss was assumed for patients with CPC 3–5 outcome and calculated for the productive ages of 55–65 years, considering the OHCA distribution by age of (0.02 for age 0–17 years, 0.04 for age 18–34 years, 0.13 for age 35–49 years, 0.3 for age 50–64 years, 0.29 for age 65–75 years and 0.22 for age > 80 years) [54]. A uniform distribution within classes was assumed. Each productive year lost was appraised by the gross domestic product per capita in Belgium of 38.5 k€ y^−1^ [55].

#### Healthcare expenditures

Outcome-associated cost of care after OHCA has been estimated by Moran et al. for Ireland [20]. Accurate recent data did not seem publicly available for Belgium. In 2016, the national expenditures on healthcare were 4.2 k€ inhabitant^−1^ vs 3.7 k€ inhabitant^−1^ for Ireland and Belgium, respectively [56]. The inflation rate over the period 2016–2018 was 6.5% for Belgium [57]. The data provided by Moran et al. was considered the most accurate at this point to estimate Belgian healthcare expenditures after OHCA for 2020 and shortly following years.

#### EVapp

Costs directly related to the implementation of EVapp are costs for insurance of the volunteers during the interventions (estimated 1% of the total population) and fixed costs for software development and maintenance, connection to the EMS dispatch systems, servers and general operating. The costs are charged to the municipality or city on the basis of the number of inhabitants (representative for the number of volunteers). The projection for Belgium amounted to 850 k€ y^−1^. Since specifically trained volunteers are recruited, the costs of training of the volunteers are costs already incurred. The cost projection related to the recruitment of the volunteers was negligible.

### Model validation and pilot case in the city of Hoogstraten

**Fig. 2:**
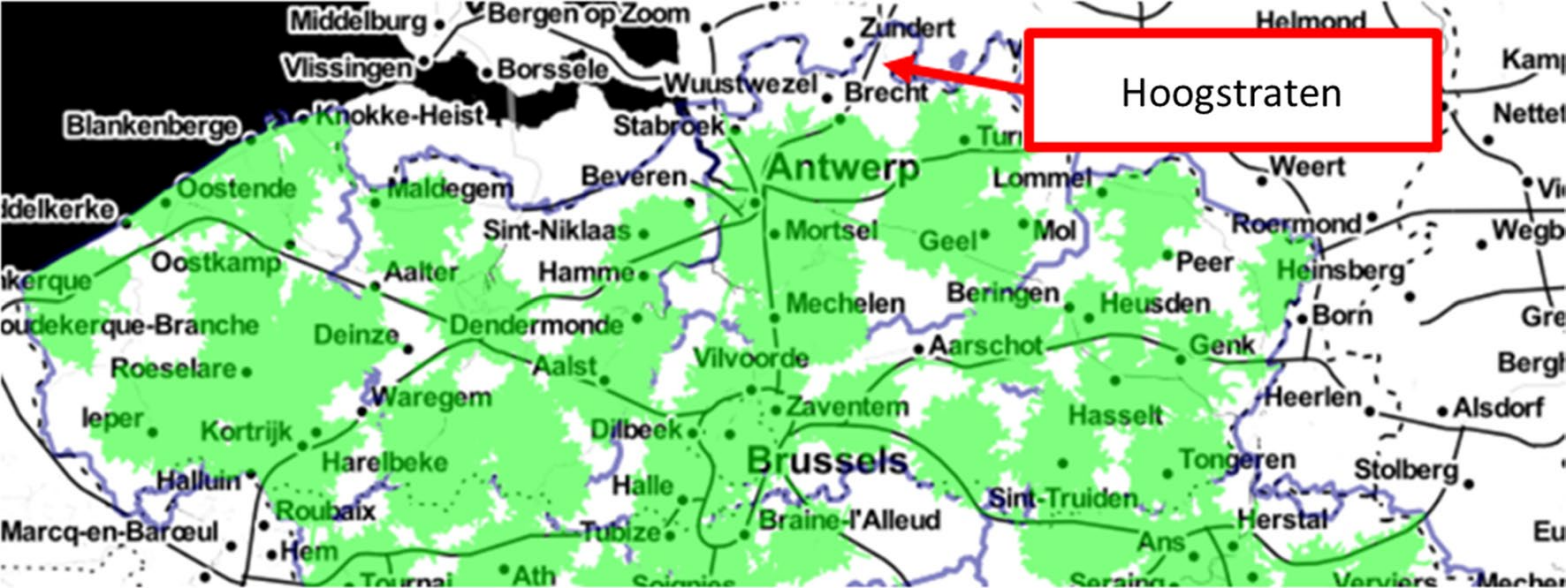
Overview of ‘blind spots’ (white background), where a mobile team of emergency physicians and nurses cannot reach the site of alert within 15 min to apply advanced life support. The upper region of Belgium (Flanders) is shown (taken from News4Med.com)

The first region in which EVapp was implemented (01/03/2017) was Hoogstraten, a city in the Belgian province of Antwerp, at the border with The Netherlands. Implementation of EVapp in Hoogstraten was selected as pilot project to compensate for its generally lower EMS and ALS coverage (‘black spots’ where a mobile team of emergency physicians and nurses cannot reach the site of alert within 15 min) (Fig. 2) [58] and lower population density of approximately 200 inhabitants km^−2^ [59].

All publicly available AED in the city were localized and registered in the EVapp database. This included the placement of new devices at the initiative of the city of Hoogstraten and efforts to make private AED’s publicly accessible.

## Results

### Simulations of outcome and cost-effectiveness

The input values used for estimation of outcomes are summarized in Tables 2, 3 and 4.

**Table 2:** Estimation of survival to discharge and neurological outcome after witnessed OHCA of cardiac origin, depending on the type of first response

%	EMS			bCPR			bCPR + AED		
	Worst	Medium	Best	Worst	Medium	Best	Worst	Medium	Best
Survival to discharge	7.8	9.4^*^	11.0	10.5	14.9*	19.3	18.2	23.2	72.0
Deceased	92.2	90.6	89.0	89.5	85.1	80.7	81.8	76.9	28.0
CPC 1–2	6.6	8.0	9.4	9.0	12.7	16.4	15.5	19.7	61.2
CPC 3–4	1.2	1.4	1.7	1.6	2.2	2.9	2.7	3.5	10.8

Data was retrieved from literature. With EMS: first response by the emergency medical system, bCPR: early bystander/first responder CPR before arrival of the EMS, bCPR + AED: early bystander/first responder CPR and defibrillation before arrival of the EMS, CPC: cerebral performance category as indication of neurological outcome (1–2: good, 3–4: bad)

*The medium case was calculated as the average value of both extreme values

**Table 3:** Estimation of the response distributions for the baseline scenario (estimated current situation in Belgium) and scenario of nation-wide EVapp implementation

Distribution	EMS	bCPR	bCPR + AED
Baseline scenario	0.64	0.33	0.03
EVapp scenario	0.25	0.50	0.25

Data was retrieved from literature

**Table 4:** Estimation of the overall outcomes after witnessed OHCA of cardiac origin for the baseline scenario (estimated current situation in Belgium) and scenario of nation-wide EVapp implementation

(%)	Worst			Medium			Best		
Baseline scenario	EMS	bCPR	bCPR + AED	EMS	bCPR	bCPR + AED	EMS	bCPR	bCPR + AED
Survival to discharge	5	3	1	6	5	1	7	6	2
Deceased	59	30	2	58	28	2	57	27	1
CPC 1–2	4	3	0	5	4	1	6	5	2
CPC 3–4	1	1	0	1	1	0	1	1	0

EVapp scenario									
Survival to discharge	2	5	5	2	7	6	3	10	18
Deceased	23	45	20	23	43	19	22	40	7
CPC 1–2	2	4	4	2	6	5	2	8	15
CPC 3–4	0	1	1	0	1	1	0	1	3

Data was retrieved from literature. With EMS: first response by the emergency medical system, bCPR: early bystander/ first responder CPR before arrival of the EMS, bCPR + AED: early bystander/ first responder CPR and defibrillation before arrival of the EMS, CPC: cerebral performance category as indication of neurological outcome (1–2: good, 3–4: bad). The values were obtained by combination of outcomes (Table 2) and response distributions (Table 3)

*The medium case was calculated as the average value of both extreme values

**Fig. 3:**
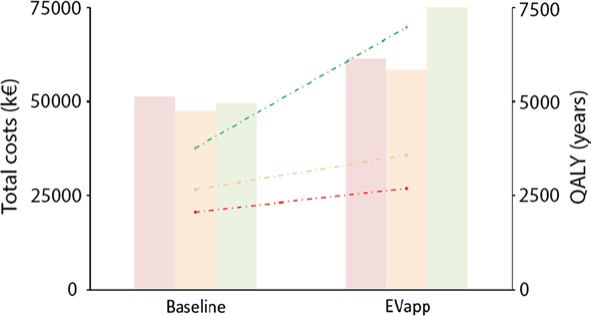
Estimation of cost of care, productivity loss and costs related to EVapp implementation (left axis) versus amount of QALYs gained (right axis) for the current baseline scenario in Belgium, compared to the scenario of nation-wide EVapp implementation in Belgium, following a 1 year cohort of witnessed OHCA victims during the average long-term survival after OHCA of approximately 6 years

As a consequence of the increased survival, costs of care were increased in the EVapp scenario compared to the baseline scenario. The total costs and productivity loss for the baseline scenario in Belgium, following 6 years of long-term mean survival after OHCA, was 47,550 k€, compared to 58,540 k€ for the EVapp case (worst case: 61,290 k€; best case: 76,515 k€) (Fig. 3). The worst case scenario was calculated using the highest range of treatment costs combined with the lowest survival. The costs related to EVapp implementation only amounted to 1.1–1.5% of the total costs. It was estimated that the amount of QALY gained by implementation of EVapp, following 6 years of long-term mean survival after OHCA, was up to 910 QALY (worst case 630; best case 3200) (Fig. 3). This means a gain of around 35%, as the estimated amount of QALY for the baseline condition was estimated at around 2670 QALY. The cost per QALY for the baseline scenario was 18 k€ QALY^−1^ (worst case 13, best case 25 k€ QALY^−1^), for the EVapp scenario these were 17 k€ QALY^−1^ (worst case 11, best case 23 k€ QALY^−1^).

### Model validation and pilot study in the city of Hoogstraten

From 1/03/2017 to 20/12/2019, the EVapp-protocol was activated 20 times by the 112-dispatcher for (suspected) OHCA, which was a lower activation rate than what would be expected from historic data suggesting 18–19 cardiac arrests year^−1^ (Federal evaluation document, EVapp-project Hoogstraten, Haenen W, personal communication). Efforts were made to identify possible causes. No abnormal under-activation of the EVapp-system in the emergency dispatch centre could be found (1 case). Yet, there was a concomitant under-activation of EMS in generally compared to other regions in the province: the number of emergency activations per 10,000 citizens was 40.7 in the city of Hoogstraten compared to 55 in the entire province. A similar observation was found in the activation of pre-hospital MUG-intervention teams that included a doctor (9.8 per 10,000 in Hoogstraten compared to 10.8 for the whole province). Although this partly accounts for the relatively low activation rate, a more comprehensive explanation on such lower rates in Hoogstraten was not available at this point. A total of 97 volunteers (around 0.5% of the population) was reached soon after the launch of EVapp (July 2017) via different local recruitment strategies. From the moment this level of 0.5% was reached, at least one volunteer responded an alert in all EVapp activations. From September 2018, 0.7% of the population was enrolled in the system. From that moment onwards, at least two volunteers responded to EVapp-activation. Both percentages are concluded to be critical numbers in adequate volunteer coverage in a rural area. At the end of the initial test phase in December 2019, 277 volunteers skilled in BLS were member of the EVapp network in Hoogstraten, which corresponded to around 1.3% of the population and to an area density of 2.6 volunteers km^−2^. The city administration decided to extend the implementation for at least another 5 years in 2020. The amount of volunteers had increased to almost 280 and consisted of both professional caregivers as well as trained lay-person (Table 5). The amount of publicly available AED in December 2019 was 19, which corresponded to approximately 0.9 per 1000 inhabitants and 0.2 AED km^−2^. Based on the performance reports generated after each activation, starting from early implementation, the EMS had not arrived before a volunteer arrived in 20% of cases and in 30% of all cases, an AED was brought to the victim by volunteers (with a clear increased trend of higher numbers achieved in the latest alerts). Negative feedback reported by the volunteers was the following: (1), insufficient indication of the location (15% of cases) and (2), the wish to receive EVapp alerts also when mobile phones were in silent mode. Both problems were further acted upon during the pilot study. All volunteers reported that they would like to remain member of the EVapp network after attending an OHCA.

**Table 5:** Overview of the 277 volunteers enrolled in the EVapp system in Hoogstraten in August 2020, by profession or as lay-person

Profession	%
Paramedic	20.5
Fireman	10.1
Volunteer red cross	10.1
Nurse	9.2
Health & safety officer	5.4
Medical doctor	3.6
Rescuer	2.5
Police officer	1.4
Volunteer (other)	37.2

## Discussion

In this research, an accessible model was made to estimate the cost-effectiveness and clinical benefits from implementation of a mobile phone application to mobilize first responders after OHCA in Belgium. Via this application, BLS-skilled volunteers in the nearest vicinity of 1500 m around the victim are activated and mobilized to apply CPR and if possible, defibrillation by public AED. The intention of the application is that the interventions would take place within 8 min post-collapse. Critical data to accurately estimate cost-effectiveness of civilian-based resuscitation initiatives are currently lacking for Belgium [2]. In absence of such data, the literature was screened to retrieve potentially relevant figures from previous initiatives. The amount of studies finally selected was rather limited, due to the large heterogeneity, absence of clear specifications on the time-delay in which interventions had taken place and the still rather limited number of operational smart phone applications. Nevertheless, the estimated overall outcomes for both scenarios can be evaluated in a broader sense (*i.e.,* baseline scenario in different countries without civilian-based systems or different PAD programs).

When civilian-based initiatives are not implemented, low values for survival after OHCA of around 10% are generally observed [1, 3, 21, 60–62]. The latter value is comparable to the estimated baseline case for Belgium (9–16%), where bystander or first-responder CPR before arrival of the EMS was considered for 33% of witnessed cases. High survival rates of 30–70% have been reported in recent studies in case of early defibrillation [9–14]. Although some of these values were for the particular case of witnessed OHCA and shockable rhythm, the overall survival rates for EVapp implementation of 12–30% for witnessed OHCA are very plausible, considering that overall survival rates of 20–50% have been observed or projected in other recent civilian-based systems [13, 28, 29, 38, 63]. Other mobile phone applications to mobilize volunteers to OHCA victims have been reported or are in development [25–29, 64]. Most data is available for the country of The Netherlands and the capital of Stockholm [26, 28, 65]. In the examples of Stockholm, activated volunteers arrived at the scene in 58% of cases [26]. The population density, percentage of volunteers and number of AED/ 1000 inhabitants were comparable to Belgium. The average distance to OHCA via AED pick-up was 1280 m, for an AED accessibility of approximately 2.5 AED km^−2^. For the rural area of Hoogstraten, different to the area surrounding the capital city of Stockholm, the AED accessibility was only 0.2 AED km^−2^ (0.9 AED/ 1000 inhabitants), and yet an AED was brought to the victim in 30% of cases during the phase of early implementation. The number of AED/ 1000 inhabitants in Belgium was considerably lower than for The Netherlands, where approximately around 5.7 AED/ 1000 inhabitants were available, of which 1.3/ 1000 inhabitants were registered at the end of 2019 [31, 66]. For a 6-min zone to function optimally, it was suggested that at least around 1.7 AED/ 1000 inhabitants should be available [66]. Sondergaard et al. investigated how the route distance to the nearest accessible AED was associated with probability of bystander defibrillation in public and residential locations by use of the nationwide Danish Cardiac Arrest Registry [67]. They found that the probability of bystander defibrillation decreased rapidly within the first 100 m route distance from OHCA to the nearest accessible AED in public areas, whereas the probability of bystander defibrillation was low for all distances in residential areas. The importance of such observations needs to be further addressed with regard to nation-wide implementation of EVapp.

Once operational, EVapp was estimated to lead to a comparable cost per QALY compared to the baseline scenario. The latter was based on a conservative increase of survivors of witnessed OHCA of cardiac origin of around 15% for the best case scenario. From an economic point of view, EVapp implementation lead to a cost of 17 k€ QALY^−1^ × 3576 QALY year^−1^ or 59 million euro year^−1^ including EVapp costs, compared to 18 k€ QALY^−1^ × 2668 QALY year^−1^ or 47 million euro year^−1^ for the baseline case scenario. These estimates underline the special cost-efficiency of EVapp to gain considerable QALY via a better use of yet available resources. Andersen et al. extensively evaluated the sensitivity and cost-effectiveness of PAD in the US [19]. They demonstrated a cost of around 50 k€ QALY^−1^ from a societal perspective and 12 k€ QALY^−1^ from a healthcare perspective. As the primer aim in this research was to increase the efficient use of yet available resources (public AED and BLS-trained volunteers), the cost of 17 k€ QALY^−1^ derived in this study is comparable to the healthcare perspective.

This study has several limitations. Literature data was used to estimate potential survival benefits and cost-effectiveness of a novel application to activate volunteers after OHCA in Belgium, based on similar initiatives and other regions. Since the system is new and regions differ in important characteristics that determine outcome of OHCA and success of community-based systems, the available literature can only provide a good estimate. The aim of this study was not to provide an inclusive overview of previous initiatives, but yet to make a fair first estimate of potential performance and costs. Studies were included in the model for which the comparator resembled the baseline scenario for Belgium or a very similar initiative was enrolled (*i.e.,* SMS-based volunteer recruitment). Assumptions needed to be made when including previous data in the current model. Such limitations was attempted to be mitigated by evaluating a worst, medium and best case scenario, by early validation of the assumptions through a pilot study, and by discussing the results of this model in the light of a plethora of previous initiatives. Within these limitations, the estimated results were found to be plausible. A recent systematic review and meta-analysis of the clinical benefits of first responder/ bystander defibrillation reported that 77% of the 44 retrieved observational studies had a critical risk of bias [8]. The quality of the evidence was low for randomized trials and very low for observational studies, data of both types of studies have been included in this research. Cost-effectiveness of community-based OHCA-interventions was found to be most strongly influenced by the incidence of OHCA, the estimated effectiveness and the actual use of public AED [8]. Such data are currently not extensive and accurate available for Belgium. Nevertheless, the first validation of the estimates for Belgium was performed by effective implementation of EVapp in the city of Hoogstraten in Flanders. The initial results carefully confirmed that the objectives and potential of EVapp may indeed be effectively achieved in practice. Further validation of the model will be carried out as EVapp is further being enrolled in various rural and urban locations in Belgium.

## Conclusions

In this study, it was estimated that nation-wide implementation of EVapp, a novel smartphone application to mobilize trained volunteers to nearby OHCA victims, would increase survival without major increase in costs. According to the best case estimates, the increase in survival for witnessed OHCA of cardiac origin was projected at 15% over the baseline scenario. This considerable increase in survival was not associated with a major increase in cost per QALY. The projections were made by use of literature data. At the moment, EVapp is being implemented in different regions in Belgium and a first successful implementation was confirmed within the rural city of Hoogstraten, a region with low population density. Further research will allow to further validate the modelled results, including different rural and urban regions.

## Data Availability

The datasets used and/or analysed during the current study are available from the corresponding author on reasonable request.
